# A non‐inferiority clinical trial comparing probiotics and oral corticosteroids for the management of acute exacerbation of atopic dermatitis patients

**DOI:** 10.1002/ski2.373

**Published:** 2024-04-06

**Authors:** Nahla A. Gamal, Mohammed A. Shoaib, Azza G. Farag, Richard Stark, Simon Tso

**Affiliations:** ^1^ Department of Dermatology Menoufia University Shebin Elkom Egypt; ^2^ South Warwickshire NHS Foundation Trust Warwick UK; ^3^ Bioinformatics Research Technology Platform University of Warwick Coventry UK

## Abstract

A prospective controlled pilot study on the feasibility of utilization of a probiotic mixture for management of acute exacerbation of atopic dermatitis (AD). Patients were allocated to either standard of care (SOC) therapy with tapering dose of steroids or a probiotic mixture over 3 weeks. After the 3‐week intervention, patients on steroids achieved significantly higher clinical response rates and significantly deeper response as measured by the change in SCORAD score. No gut microbiome changes could be appreciated in either group after the treatment period. We could conclude that probiotics cannot replace SOC therapy for the management of acute exacerbation of AD.

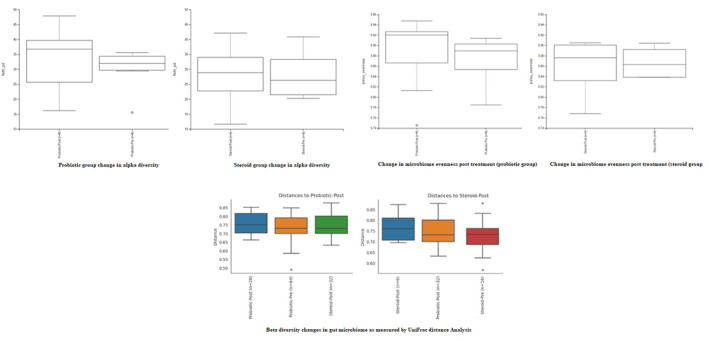

## CONFLICT OF INTEREST STATEMENT

All authors have no conflicts to declare.

## AUTHOR CONTRIBUTIONS

**Nahla A. Gamal**: Data curation (equal); formal analysis (equal); methodology (equal); project administration (equal); writing – original draft (equal); writing – review & editing (equal). **Mohammed A. Shoaib**: Funding acquisition (equal); project administration (equal); supervision (equal). **Azza G. Farag**: Project administration (equal); supervision (equal). **Richard Stark**: Data curation (equal); formal analysis (equal); software (equal); writing – review & editing (equal). **Simon Tso**: Project administration (equal); resources (equal); supervision (equal); validation (equal); writing – original draft (equal); writing – review & editing (equal).

## FUNDING INFORMATION

Ministry of Higher Education (Egypt) and British Council

## ETHICS STATEMENT

The study (with its different phases) has been approved by the ethics and research committee at Menoufia University Hospitals, Egypt, according to the research policy of the ministry of high education and scientific research in Egypt. Patient recruitment for the study started only after getting full ethical approval. All patients (and their legal guardians where applicable) were consented to the study and publication of the results once obtained.

Dear Editor,

Atopic dermatitis (AD) has been well recognized for its major health burden, which seems to be proportional to gross domestic product, affecting up to 20% of children and 10% of adults.^1^ Extensive research revealed a complex aetiopathogenesis of the disease, where gut microbiome would play an underpinning role into the development and course of the disease.^2^, ^3^ Gut dysbiosis, as shown by lower diversity, increased pathobionts and decreased relative abundance of short chain fatty acid producers, has been reproducibly demonstrated in patients with AD.^4^, ^5^

In view of the chronic relapsing nature of the disease (and subsequently the need for repetitive courses of topical and systemic therapies with their recognized local and systemic adverse effects),^6^ there has been a long pursuit for safe and inexpensive alternative therapeutic options. In this context, targeting gut microbiome, as a driver of the development and relapses of AD, has been an alluring option given the relative safety and broader health benefits of implanting health promoting species in the human gut compared to other medications.^7^ Probiotics have been utilized in many clinical trials for the management of AD with promising results.^8^ However, there has been inconsistency between different studies on the duration of treatment, probiotic strains administered, age of the recipient patients and whether probiotics were used as adjunctive therapy to steroids, which might explain the difference in clinical outcome.^9^

We conducted a prospective controlled non‐inferiority pilot study to explore the therapeutic potential of probiotics as a solo treatment in an Egyptian cohort of patients with moderate to severe AD. To the best of our knowledge, this is the first study to investigate the feasibility of utilization of probiotics as a solo treatment of acute exacerbation of AD in a head to head comparison with standard of care (SOC) therapy (steroids). After ethical approval, patients with SCORAD score ≥25 were allocated to either SOC therapy (tapering course of steroids, starting at 0.5 mg/kg) or probiotics (a mixture of 2 *lactobacilli* strains; *Lactobacillus delbruekii* and *Lactobacillus fermentum* at a dose of 1 × 10^9^ CFU twice daily) for 3 weeks. Those allocated to the probiotic group declined repetitive courses of steroids for flare ups of their disease and pursued some other form of safe natural remedies. Initially, 29 patients were enroled for the trial (15 allocated to steroid, and 14 to probiotic). Ten patients in total dropped out throughout the study course with 19 patients left to final per‐protocol analysis (11 in probiotic group and 8 in steroid group). After 3 weeks of intervention, patients were assessed for clinical improvement (as primary outcome) based on the change in SCORAD score. Patients provided stool samples at baseline and after treatment in both groups for gut microbiome assessment, via 16s DNA sequencing, to explore any underpinning microbiome changes that might contribute to potential clinical improvement (as a secondary outcome).

The use of prebiotics, other natural or synthetic probiotics, antibiotic or other systemic medications was prohibited for the trial duration. Similarly, antibiotics, probiotics and prebiotics use during the 3 months preceding the study, was an exclusion criterion to enrolment in the study, as was history of chronic diseases, other atopic conditions, or the use of proton pump inhibitors for the previous 2 weeks, given that all these factors can perturb gut microbiome and confound the results. Gut microbiome assessment was performed on a representative sample of each group (8 in probiotic group & 4 in steroid group).

After 3 weeks of treatment, all patients in the steroid group achieved clinical response compared to lower response rate in probiotic group (36.36%), (*p* value = 0.013). More importantly, the magnitude of response as defined by the percentage of a change in SCORAD score was significantly higher in the steroid group (*p* value < 0.001) Table 1. There were no statistically significant differences between both patient groups at baseline in terms of demographic characteristics. Similarly, no significant differences existed between responders and non‐responders in terms of sex, socio‐economic status, residency among other demographics.

**TABLE 1:** Baseline demographic characteristics and clinical outcome in both patient groups.

	Probiotic group (*N* = 11)	Steroid group (*N* = 8)	*χ* ^2^	*p* value
Age	8 ± 2.42	10 ± 17.27	28.500^a^	0.206
Sex (M/F)	(8/3)	(4/4)	1.028^b^	0.377
Baseline SCORAD score	46.44 ± 15.43	42.07 ± 13.67	39.500^a^	0.717
Residency (rural/urban)	(4/7)	(4/4)	0.353^b^	0.658
Socio‐economic status (high/medium/low)	(4/3/4)	(2/0/6)	3.254^b^	0.215
Pet ownership	18.19%	50%	2.170^b^	0.319
Mode of delivery (caesarean/vaginal)	(7/4)	(2/6)	2.773^b^	0.170

	Clinical outcome			
Change in SCORAD score (% decrease)	26.43 ± 0.09%	51.50 ± 0.04%	0.000^a^	<0.001
Responders within each group	36.36%	100%	8.061^a^	0.013

^a^ Mann–Whitney test.

^b^ Chi square/Fisher's exact.

**FIGURE 1:**
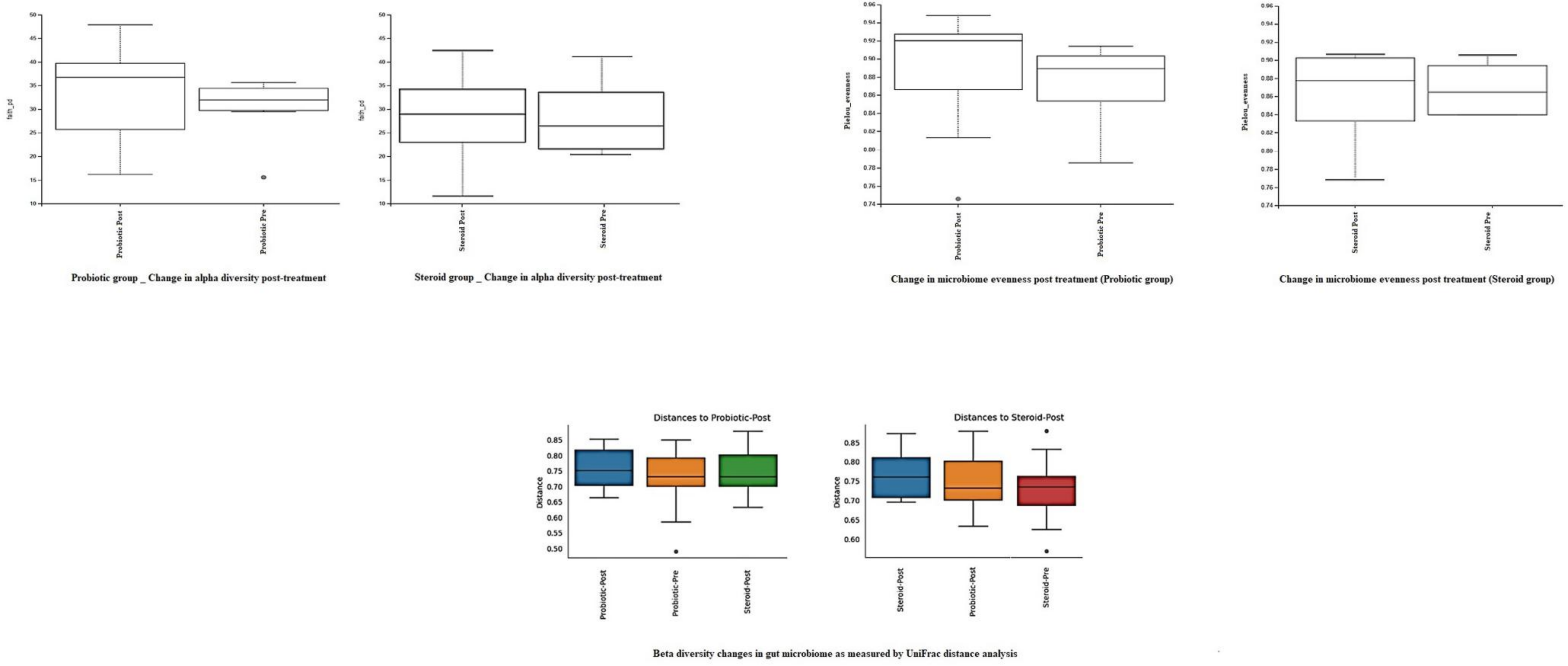
Alpha and beta diversity changes in both patient groups after treatment compared to baseline.

Sub‐group gut microbiome analysis revealed no significant changes in alpha diversity in both treatment arms after therapeutic intervention as demonstrated by Faith's Phylogenetic diversity (Kruskal–Wallis *p*‐value for all groups = 0.76), although there was a trend of increase of the mean Alpha diversity (*p*‐value [Kruskal–Wallis] = 0.25). In pars with that, unweighted Unifrac Distance analysis of β‐diversity showed no significant clustering of microbiome communities in different groups with cPermanova *p*‐value (All groups = 0.78) (Figure 1).

Although our findings contradicted many other trials which reported promising therapeutic effect of probiotics for the treatment of AD,^10^ this discrepancy could be explained by several factors including small sample size in our pilot study, using probiotics as a solo treatment in head to head comparison to steroid therapy in acute exacerbation of moderate to severe AD, along with shorter duration of treatment compared to other trials and finally using different strains of *lactobacilli* (based on availability in the Egyptian market). Our study was one of a few which investigated gut microbiome changes as an underpinning mechanism to any potential therapeutic effect. In concordance with the negative clinical outcome of probiotic treatment, we could not identify any considerably positive gut microbiome change post‐treatment. This emphasizes the need for a positive change in gut microbiome ecosystem for probiotics to produce any effect. Our study has limitations. This is a small pilot study with a higher drop‐out rate in the probiotic group compared with oral corticosteroids group potentially due to the lower efficacy of probiotics. Our study could not identify characteristics of subgroups that are more likely to benefit from probiotics. We could finally conclude that probiotics cannot be used as a solo treatment for management of acute AD, and where administration of probiotics is considered, it should be for prolonged time and as an adjunctive or maintenance therapy. In addition, where probiotics are to be expected to provide any additional benefit in AD, this has to be in the context of a favourable gut microbiome change as concluded from our study and other previous contradictory studies.

## Data Availability

The data that supports the findings of this study are available in the supplementary material of this article.
